# PET/CT with Fluorodeoxyglucose During Neoadjuvant Chemoradiotherapy in Locally Advanced Rectal Cancer

**DOI:** 10.3332/ecancer.2016.629

**Published:** 2016-03-29

**Authors:** Laura L Travaini, Maria G Zampino, Marzia Colandrea, Mahila E Ferrari, Laura Gilardi, Maria C Leonardi, Luigi Santoro, Roberto Orecchia, Chiara M Grana

**Affiliations:** European Institute of Oncology, Via Ripamonti, 435 20141, Milan, Italy

**Keywords:** rectal cancer, neoadjuvant therapy, PET/CT, fluorodeoxyglucose

## Abstract

**Objective:**

The aim of the present study is to evaluate the accuracy of Positron Emission Tomography/Computed Tomography (PET/CT) with Fluorodeoxyglucose ([18F]FDG) to predict treatment response in patients with locally advanced rectal cancer (LARC) during neoadjuvant chemoradiotherapy.

**Patients and methods:**

Forty-one LARC patients performed [18F]FDG-PET/CT at baseline (PET0). All patients received continuous capecitabine concomitant to radiotherapy on the pelvis, followed by intermittent capecitabine until two weeks before curative surgery. [18F]FDG-PET/CT was also carried out at 40 Gy-time (PET1) and at the end of neoadjuvant therapy (PET2). PET imaging was analysed semi-quantitatively through the measurement of maximal standardised uptake value (SUVmax) and the tumour volume (TV). Histology was expressed through pTNM and Dworak tumor regression grading. Patients were categorised into responder (downstaging or downsizing) and non-responder (stable or progressive disease by comparison pretreatment parameters with clinical/pathological characteristics posttreatment/after surgery).

Logistic regression was used to evaluate SUVmax and TV absolute and percent reduction as predictors of response rate using gender, age, and CEA as covariates. Progression-free survival (PFS) and overall survival (OS) were estimated by the Kaplan-Meier method. Survivals were compared by the Log-Rank test.

**Results:**

Twenty-three responders (9 ypCR, 14 with downstaged disease) and 18 non-responders showed differences in terms of both early and posttreatment SUVmax percent reduction (median comparison: responder = 63.2%, non-responder = 44.2%, p = 0.04 and responder = 76.9%, non-responder = 61.6%, p = 0.06 respectively). The best predictive cut-offs of treatment response for early and posttreatment SUVmax percent reduction were ≥57% and ≥66% from baseline (p = 0.02 and p = 0.01 respectively).

**Conclusions:**

[18F]FDG-PET/CT is a reliable technique for evaluating therapy response during neoadjuvant treatment in LARC, through a categorical classification of the SUV max reduction during treatment.

## Introduction

Locally advanced rectal cancer (LARC) is a clinical condition staged for tumour involvement in the rectal wall and regional lymph nodes with rectal endoscopic ultrasonography (EUS), computed tomography (CT), and magnetic resonance imaging (MRI).

The standard treatment is neoadjuvant therapy followed by radical surgery. Because of the demonstrated clinical advantage when compared with surgery alone, it has been reported that the neoadjuvant approach with chemoradiotherapy (CRT) reduces tumour size and induces downstaging [1, 2], and also it increases resectability and sphincter conservation with a lower rate of acute toxicity and local relapse [3, 4].

Despite the complexity of the treatment, pathological complete response (ypCR) as ypT0ypN0 is achieved by only 15–20% of patients (pts), and it is to be noted that pts with ypCR seemed to have a better outcome [5, 6].

Therefore, the opportunity to predict ypCR status during the course of or at the end of treatment could lead to a potentially less aggressive surgery or avoidance of surgery altogether in selected cases.

Current standard techniques performed at baseline, such as EUS, CT, and MRI do not provide reliable results useful to predict response because of their under- or overestimation of local tumour extension [7, 8]. By contrast some studies have demonstrated that PET with fluorodeoxyglucose ([18F]FDG) are able to provide early information about metabolic response, which can further be correlated with histopathological status [9–15].

[18F]FDG-PET may also be useful for identifying neoadjuvant treatment response thereby allowing to potentiate a prompt systemic treatment or RT (i.e. a boost to the primary tumour or the positive node) in nonresponsive patients within the concept of a personalised care strategy.

The goal of this study is to further investigate the accuracy of [18F]FDG-PET in assessing treatment response during neoadjuvant therapy in a series of 41 pts with LARC.

## Patients and methods

### Patients

Between October 2007 to December 2009, we undertook a prospective study enrolling 63 pts affected by LARC, T3–T4/N0 or any T with positive regional nodes, M0 were candidates for neoadjuvant CRT followed by radical surgery. Eligibility criteria included: histologically proven adenocarcinoma, age ≥18 years; Eastern Cooperative Oncology Group (ECOG) performance status ≤2; no prior chemotherapy or RT to the pelvis; life expectancy ≥3 months; adequate haematologic, liver, and renal function (granulocyte count >1500/mm^3^, platelet count >100,000/mm^3^, haemoglobin >10 g/dL, serum creatinine <1.25 ULN, bilirubin <1.25 ULN, AST or ALT <2 ULN). Patients without compensated concurrent pathologies were excluded.

At baseline, a surgical evaluation with digital rectal examination was performed. Subsequent assessment included colonoscopy with tumour biopsy, complete history and physical examination, chest and abdominopelvic CT scan, and routine blood tests.

Forty-one out of 63 pts underwent [18F]FDG-PET/CT at baseline (PET0): the mean time between start of therapy and PET0 was five days (range 5–43).

PET was also performed at either 40 Gy-time, which corresponds to the 22nd day after the onset of the preoperative treatment (PET1), and at completion of CRT (PET2): the mean time between the end of neoadjuvant therapy and surgery was eight weeks (range 7–9 weeks).

The local stage was determined according to World Health Organisation criteria by rectal EUS, and in the case of stenotic tumour unavailable by endoscopy, by pelvic MRI, or CT scan. Positive nodes were identified by specific morphologic and dimensional criteria through the RECIST criteria [16]. Patients and tumour characteristics at baseline are reported in Table 1.

Based on clinical staging, a proposal of surgery was strategised: either low anterior or through Miles abdominoperineal or coloanal resection. Patients with obstructive lesions underwent temporary colostomy before the start of the study. All patients gave written informed consent for study procedures.

### Treatment protocol

Preoperative radiotherapy was delivered by using a high-energy linear accelerator (≥15 MV). All patients were treated in the prone position, with a device to displace the small bowel (belly board mainly). The whole pelvis was treated with the four-field technique up to 45 Gy in 25 fractions over five weeks.

The boost volume (the mesorectum surrounding tumour and involved nodes) was irradiated by multiple conformal fields delivering 5.4 Gy in three fractions.

Chemotherapy with capecitabine at 825 mg/m^2^ was continuously delivered twice a day orally, concomitant to radiotherapy. After one to two weeks rest period, one to two full cycles of capecitabine 1000–1250 mg/m^2^ twice a day, two weeks on and one week off, were given until at least one to two weeks before curative surgery. To maximise tumour regression, surgery was performed within ten weeks from the end of RT after clinical and instrumental tumor reassessment. A sphincter saving procedure was proposed when the lesion was at least 3 cm from the anal verge, with no signs of infiltration of the external sphincter or of the pelvic floor.

In pts who obtained ypCR and/or in the case of pts with downstaging, downsizing (tumour shrinkage without downstaging), or stable disease, chemotherapy with fluoropyrimidine was performed up to a complete overall duration of six months. For pts with pathologic nodal involvement, oxaliplatin adjuvant combination was considered.

In this scenario, responder patients were identified by downstaging or downsizing (tumour shrinkage without downstaging) whereas non-responder by stable or progressive disease, and also by comparison of pretreatment parameters with clinical/pathological characteristics posttreatment/after surgery.

### Pathological analysis

In all cases routinely formalin-fixed and paraffin embedded and E & E-stained sections were used. In the histological report T status, tumour type, occurrence of vascular (classified as intramural and extramural venous vessels or lymphatic channels) and perineural invasion, Dworak tumour regression grade (TRG), number of metastatic lymph nodes (three levels for every lymph node) were described.

Tumour regression after CRT was evaluated with the semiquantitative five-point tumour Dworak TRG system [16] evaluating histological changes in the tumour. This classification identifies several different grades of TRG ranging from no regression to complete disappearance of tumour cells: TRG0 as no regression, TRG1 as minor regression with fibrosis in only 25% or less of the tumour mass, TRG2 as dominant tumour mass with obvious fibrosis in 26–50% of the tumour mass, TRG3 as dominant fibrosis outgrowing the tumour mass, and TRG4 as total regression, total fibrotic mass, and no viable tumour cells.

If the ypCR as ypT0 ypN0 were achieved, the whole lesion was paraffin embedded and three sections for every block were further cut.

### Statistical analysis

Statistical analysis includes descriptive statistics (median and range for continuous variables; percentage values for categorical variables). Absolute reduction for early (overall) SUVmax and tumour area reduction were calculated as the difference between pretreatment and on-treatment (posttreatment) values; percentage reduction were calculated by dividing the absolute reduction by the pretreatment value and multiplying the result by 100. Univariate logistic regression models were used to evaluate the ability of early (overall) absolute (percent) reduction of SUVmax (tumour area), as continuous variables, in predicting treatment response. Once selected the variables significantly associated to treatment response, their areas under the receiver operating characteristics curves (AUCs) were estimated to determine the cut-off discriminating between best responder and non-responder. Sensitivity (Se), specificity (Sp), positive and negative predictive values (PPV, NPV) of the new binary variables were calculated. The value of the dichotomised variables as independent predictors of the response rate was evaluated by multivariate logistic regression models.

Gender, age, and CEA (<=5 versus >5) were used as covariates. PFS and OS were estimated by the Kaplan-Meier approach and survivals were compared by the Log-Rank test. A difference with a p value < 0.05 is considered statistically significant.

### FDG-PET/CT

Patient preparation, imaging, and reconstruction protocols were kept constant for serial scans in the same patient. After at least six hours of fasting, pts were injected with 3 MBq/kg body weight of [18F]FDG. Fasting serum glucose levels were measured 10 to 15 minutes before [18F]FDG administration. It was found that all measured values were less than 200 mg/dL (the mean value was 170 mg/dL).

Whole-body imaging (WB) was carried out in all patients after a mean uptake period of 50 min on a GE Discovery ST PET/CT scanner (GE Healthcare, Milwaukee, Wisconsin, USA) using a standard skull base to pelvis protocol.

Acquisition was performed with patients lying supine on the scanner bed with their arms raised above their head.

The CT acquisition protocol included a low-dose CT (120 kV, 80 mA, 0.8 seconds per rotation, pitch 1.35, 3.75-mm slice thickness) from the base of the skull to mid-thigh for attenuation correction followed by the WB PET scan (3 min per bed position). After the baseline acquisition, a dedicated PET static emission images of the tumour region was acquired for the RT treatment planning.

The PET scans were acquired in 3D mode in a 256 × 256 matrix. Images were reconstructed by the Vue point attenuation weighted ordered-subsets expectation maximisation (OSEM) algorithm (two iterations, 30 subsets) followed by a postreconstruction smoothing Gaussian filter (4.5 mm full width at half maximum). PET scans were interpreted by semi-quantitative measurement (SUVmax): the measurement of SUV normalised to the injected dose and to body weight (SUVbw max) was always conducted and collected.

Using the Advantage Windows workstation (GE Healthcare), the tumour region was identified. SUVbw max was determined in the tumour region and used to define the tumour volume (TV) through a threshold level of 42% of the SUVmax. The metabolic response was assessed in terms of reduction in SUVmax among the three PET/CT scans.

## Results

[18F]FDG-PET/CT revealed increase tracer uptake in the primary tumour in all patients. The median SUVbw max of the rectal lesions was 25.3 (9.9–99.5) at PET0, 11.1 (6.0–28.5) at PET1, and 7.0 (1–30.0) at PET2 (Table 2).

The median TV measured by PET was 20.0 mL (3.5–94.1) at PET0, 4.6 mL (1.2–45.5) at PET1, and 2.4 mL (1.0–15.8) at PET2. In 20 pts the TV was <1 at PET2 (Table 2).

The estimated lymph nodal involvement revealed by [18F]FDG-PET/CT was always confirmed by at least, one of the other imaging techniques.

In four pts, PET/CT identified increased [18F]FDG uptake outside the pelvic region (two in the liver and two in the lung parenchyma) suspected as metastatic disease, and in all four cases the site was always confirmed by conventional imaging (CI) in a subsequent phase.

Regarding the primary tumour, the PET1 scan was positive in 74.4% (17 true positive), doubtful in 21%, and negative in 4.6% (two true negative). PET2 scan was positive in 28.6% (two false positive), doubtful in 28.6%, and negative in 42.8% (11 true negative).

Of the 16 pts with TRG 3 and 4 (Table 3), all were in ypCR and only one was ypN+, meanwhile of the 25 pts with TRG 1 and 2 the TNM was in agreement in 76.0% (19/25).

**Figure 1.**
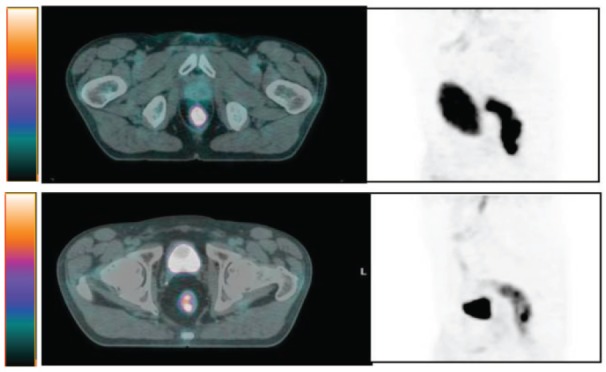
Example of non-responder patient (G.F. cT3N0. SUVbw max at PET0 65. SUVbw max at PET2 30. Histopathological analysis ypT3pN1 (regression index 1).

**Figure 2.**
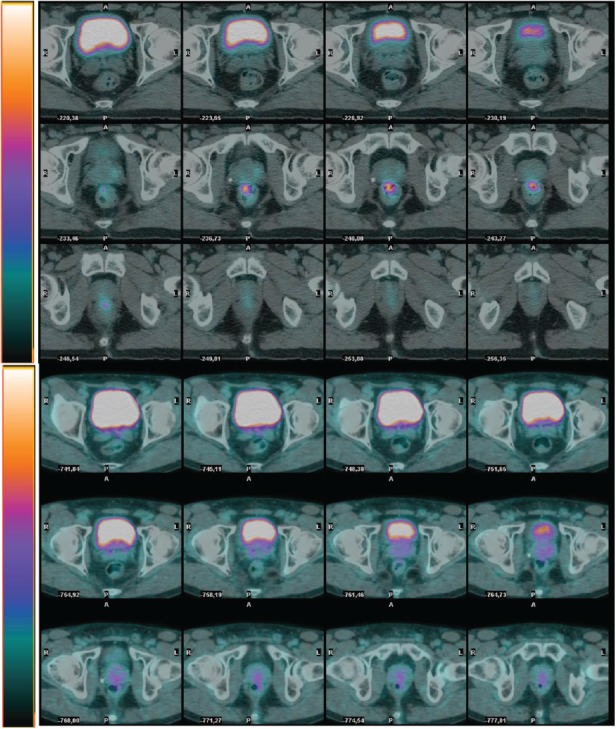
Example of responder patient (C. V.cT2N0 SUVbw max at PET0 15.6. SUVbwmax at PET2 5. Histopathological analysis ypT0N0 (regression index 4).

Twenty-three responder (R), 9 pts with ypCR and 14 with downstaged disease and 18 considered non-responder (NR) because of downsizing or stable disease, showed a significant difference in terms of early SUVmax percent reduction (median comparison: NR = 44.2%; R = 63.2%; p = 0.04) and a difference in terms of posttreatment SUVmax percent reduction which was not statistically significant (median comparison: NR = 61.6%; R = 76.9%; p = 0.06). On the contrary no difference was found in terms of early and post-treatment SUVmax absolute reductions, as well as in terms of early and post-treatment TV reduction (Table 2). In Figures 1 and 2 we show two examples of R and NR pts.

**Figure 3.**
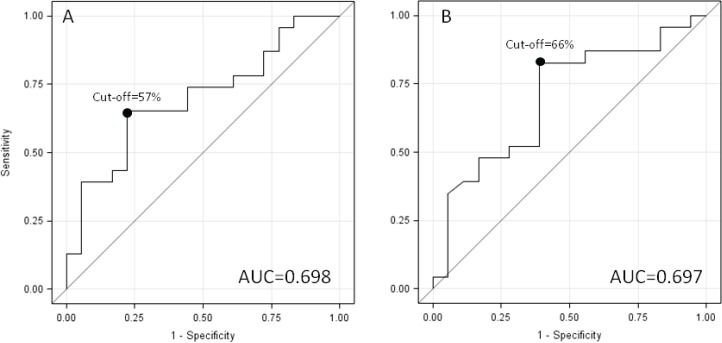
ROC Curves for SUVmax early and late reduction.

The categorical classification of the SUVmax reduction during treatment (at least 57% from baseline) was significantly predictive of therapy response (P = 0.009 in univariate analysis, p = 0.02 in multivariate analysis) when the response was classified by the pTNM. As a result of the cut off which was equal to 57% (Figure 3), AUC was very close to 70%, Sp and PPV were close to 80%, while Se and NPV were approximately 65% (Table 4). A cut-off of 66% for SUVmax reduction posttreatment (at least 65% from baseline), provided very similar results (Figure 3 and Table 4).

No difference in terms of TV variation over time was observed between R and NR when classified according to TRG (data not shown).

### Clinical outcome

Median follow-up was 37 months (14–56). Eight patients out of 41 relapsed (local relapse in three and distant relapse in five), two of them died. One additional patient died without evidence of oncological disease progression.

**Figure 4.**
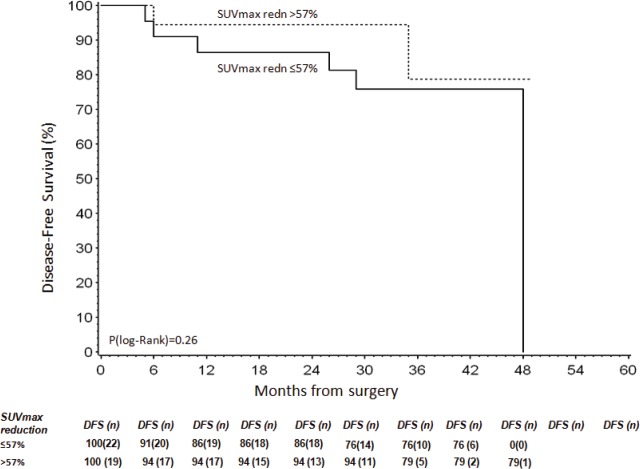
DFS according to SUVmax early reduction.

The estimated four-year PFS, 95% CI was 39% (2–81%). There were no differences in terms of four-year PFS between subgroups of patients with and without early reduction (≥57%) of SUVmax reported on-treatment versus SUVmax pre-treatment (p[log-rank]=0.26, Figure 4).

## Discussion

Although PET imaging was initially reported in late 1992 to assess therapy response in LARC pts, no consensus has yet been reached on its accuracy to discriminate patients who are R from those who are NR during neoadjuvant treatment and on the optimal timing of the PET scan evaluation [18]. This scenario has led to a growth of data with contrasting/conflicting results.

The role of [18F]FDG-PET/CT has been investigated during neoadjuvant CRT treatment at different timing from the day eight (10.8 Gy) [13] to day 14 (19.8 Gy) [9–19] and after therapy [11, 20–22]. Perez [21] and Murcia [22] reported that FDG-PET is a reliable technique for selecting patients who may avoid unnecessary radical surgery. Other authors [23–24] failed to correlate the SUVmax reduction with ypCR after neoadjuvant therapy. Conversely, Oku et al [25] demonstrated that the SUVmax of the primary tumour at the end of neoadjuvant therapy is the only prognostic variable for progression-free survival (P = 0.05). Similarly Capirci [11] reported a disease-free survival (DFS) of 93% and Murcia Duréndez [22] reported the reliability of FDG-PET in therapy response evaluation.

Our study confirms the published results [11, 26]: a percentage reduction of SUVmax of at least 57% is accurate in predicting therapy response during neoadjuvant treatment. However, the percentage of reduction in Capirci and Shanmugan papers was higher, being 66.2% and 63% respectively. The different ΔSUV we found may be explained by the different timing of PET during neoadjuvant therapy and by different chemotherapeutic agents that may be responsible for the different response of surrounding tissue. We also found that a reduction of TV (70% at least) during treatment is predictive of therapy response (multivariate p-value = 0.09, adjusted by gender, age, and CEA), compared to the TNM.

Unfortunately, no significant correlation was obtained when ΔSUV and TV were compared to TRG.

One of the major limitations of PET imaging is the parameters of evaluation: qualitative data are burdened by subjectivity, whereas semi-quantitative parameters do not consider some contingent conditions (i.e. colitis, urinary stasis). In our opinion, both methodologies should be always considered in order to obtain a more objective scenario, avoiding under- and overestimation of [18F]FDG uptake. In our population, three pts revealed increased FDG uptake outside the primary tumour, i.e. in relation to colon adenoma and colitis (toxic effect of chemotherapeutic drugs and inflammatory effect of radiotherapy) respectively.

Indeed early [18F]FDG modification [9], most of all during radiotherapy treatment as previously reported [27], can be used to detect histological peritumoural inflammation cells, which limits the predictive value of PET/CT results.

Although the [18F]FDG-PET/CT scan can be predictive of clinical and pathological outcomes in LARC, it is not free of limitations.

As reported by other authors [28], [18F]FDG-PET/CT is considered inaccurate in determining nodal involvement when compared to CI. Two of the possible reasons are the proximity of the mesorectal lymph nodes to the primary tumour, which often hides them, as it happens in an oesophageal cancer [29] and also given the limited dimensions of the mesorectal lymph nodes, these are therefore not detectable with PET [30]. The absence of histological confirmation of nodal involvement in LARC at staging prevents us from having a precise delineation of the performance of all techniques, although literature suggests that the best performance comes from pelvic MRI [31] and rectal EUS [32].

In our study [18F]FDG-PET/CT suggests lymph nodal involvement in 10/63 pts (15.9%) versus 46/63 (73%) from CI.

Moreover, if we look at the SUV of PET2 and choose five as cut-off to distinguish R from NR pts, as did Martoni [33], we found one relapse in the SUV <5 group versus eight relapses in the SUV> 5 group though the difference in the two groups was of no statistical significance (p (chi-square)=0.26. If we compare the two groups in terms of DFS, we obtain a DFS (four year) per SUV <5: 90% versus a DFS (four year) per SUV >5: 36.9%. Again the difference was not statistical significant (p(Log-Rank)=0.37.

However, in our sample the best SUV cut-off of PET2, able to best distinguish R from NR, was SUV ≤ 6 (responder: 12/18; 66.7%) versus SUV > 6 (responder 11/26; 42.3%). We found two relapses in the group of SUV ≤ 6 versus 7 relapses in the group of SUV > 6. The difference in the two groups was no statistically significant (p(chi-square)=0.20). And if we compare the two groups in terms of relapse- free survival (RFS) we obtain a RFS (four year) for SUV ≤ 6 and SUV > 6 of 85.6% and 73.8% respectively. The difference once again was not statistically significant (p(Log-Rank)=0.13).

All these results confirm the previous literature data [33] and are probably to be correlated with the lower recurrence and death rates in our population.

## Conclusions

Results from our study go towards the direction of using [18F]FDG-PET/CT as a technique for evaluating therapy response during neoadjuvant treatment in LARC. However, we were not able to totally prove the reliability of the technique, mainly because of the accuracy of the data, which can be significantly improved, and given the small sample size of the study.

## Conflict of interest

The authors declare no conflict of interest.

## Funding statement

The authors received no funding for this study.

## Figures and Tables

**Table 1.** Baseline patient and tumour characteristics.

Characteristics	Patients n (%)
**Age (years)**	
Median (min-max)	61 (40–75)
**Gender**	
Males	26 (63.4)
Females	15 (36.6)
**CEA (ng/mL)**	
<5	30 (73.2)
≥5	11 (26.8)
**cT cN**	
cT2 cN0	1 (2.4)
cT2 cN+	0
cT3 cN0	10 (24.4)
cT3 cN+	24 (58.5)
cT4 cN0	2 (4.9)
cT4 cN+	2 (4.9)
cT n.e cN+	2 (4.9)
**cN CI**	
N0	13 (31.7)
N+	28 (68.3)
**cN PET0**	
N0	33 (80.5)
N+	8 (19.5)

**Table 2.** SUVmax and TV. Absolute and % reduction.

	ALL Patients (N = 41)	Non-responder (N = 18)	Responder (N = 23)	*p-value*^^^
	*Median (min, max)*	*Median (min, max)*	*Median (min, max)*	
**SUVmax**				
PET0	25.3 (9.9, 99.5)	24.3 (9.9, 99.5)	24.6 (12.2, 61.8)	
PET1	11.1 (6.0, 28.5)	14.2 (6.0, 28.5)	9.7 (6.5, 25.1)	
PET2	7.0 (1.0, 30.0)	8.1 (2.7, 30.0)	6.0 (1.0, 15.3)	
**PET1 versus PET0**				
*Absolute reduction*	*14.7 (0.8, 74.0)*	*11.1 (0.8, 74.0)*	*17.7 (2.1, 39.4)*	*0.55*
*% reduction*	*56.2 (5.2, 82.8)*	*44.2 (5.2, 74.4)*	*63.2 (14.2, 82.8)*	***0.04***
**PET2 versus PET0**				
*Absolute reduction*	*19.8 (-8.1, 89.9)*	*16.5 (-8.1, 89.9)*	*21.7 (5.3, 46.5)*	*0.56*
*% reduction*	*74.0 (-49.1, 95.9)*	*61.6 (-49.1, 90.4)*	*76.9 (30.7, 95.9)*	***0.06***
**TV**				
PET0	20.0 (3.5, 94.1)	19.3 (3.5, 94.1)	20.9 (4.3, 77.2)	
PET1	4.6 (1.2, 45.5)	5.5 (1.2, 45.5)	4.3 (1.4, 19.6)	
PET2 [#]	2.4 (1.0, 15.8)	3.8 (1.0, 15.8)	2.1 (1.2, 15.0)	
**PET1 versus PET0**				
*Absolute reduction*	*15.0 (-11.3, 87.2)*	*12.2 (-11.3, 87.2)*	*17.2 (0.9, 62.5)*	*0.81*
*% reduction*	*75.1 (-119.8, 95.7)*	*71.7 (-119.8, 92.6)*	*77.0 (16.6, 95.7)*	*0.13*
**PET2 versus PET0 [#]**				
*Absolute reduction*	*21.1 (-6.4, 84.8)*	*19.2 (-6.4, 84.8)*	*21.7 (2.7, 68.3)*	*0.86*
*% reduction*	*87.8 (-68.0, 96.2)*	*87.0 (-68.0, 94.1)*	*91.3 (52.2, 96.2)*	*0.45*

^ Wald test from logistic regression; absolute and % reduction: continuous variables used as predictors of TNM response.

[#] PET2: Posttreatment TV≥1 for only 21 patients (11 and 10 non responder and responder respectively).

**Table 3.** Correlation between TRG and patient’s characteristics.

Parameters	TRG (3–4) (N = 16)	TRG (1–2) (N = 25)
**ypT**		
yPT3-T4	0	19
yPT0-T1–T2	16	6^*^
**ypN**		
yPN+	1	12^^^
yPN0	15	13
**SUVmax [#]**		
PET1	9.6 (7.0, 17.0)	12.5 (6.0, 28.5)
PET2	6.4 (3.8, 14.7)	7.6 (1.0, 30.0)
**TV [#]**		
PET1	4.4 (1.4, 19.6)	4.8 (1.2, 45.5)
PET2	2.1 (1.4, 15.0)	2.7 (1.0, 15.8)
**Responder TNM**		
Yes	16	7
No	0	8

* 5ypN0, 1ypN+

^ 9 ypN1

[#] median (min, max).

**Table 4.** SUVmax by responder TNM (categories of absolute and % change).

		TNM Responders								
		No [n = 18]	Yes [n = 23]	p-values		Se	Sp	PPV	NPV	AUC
	N	n	n	^	~					
**Early percent change**				0.009	0.02	65.2%	77.8%	78.9%	63.4%	0.698
≤57%^*^	*22*	*14*	*8*							
>57%	*19*	*4*	*15*							
**Overall percent change**				0.006	0.01	82.6%	61.1%	73.1%	73.3%	0.697
≤66%^*^	*15*	*11*	*4*							
>66%	*26*	*7*	*19*							

* Cutoff identified by the ROC curves;

^ Unadjusted estimate;

~ from multivariate logistic regression: estimate adjusted by gender, age and CEA (< = 5 versus >5);

Se = Sensitivity;

Sp = Specificity;

PPV = Positive Predictive Value;

NPV = Negative Predictive Value;

AUC = Area Under Curve.
